# In Search of Model Ecological Systems for Understanding Specialized Metabolism

**DOI:** 10.1128/mSystems.00175-17

**Published:** 2018-03-27

**Authors:** Rita C. Pessotti, Bridget L. Hansen, Matthew F. Traxler

**Affiliations:** a Department of Plant and Microbial Biology, University of California, Berkeley, California, USA

**Keywords:** chemical ecology, interspecies interactions, microbial ecology, microbiome, model systems, specialized metabolism

## Abstract

Microbes occupy diverse habitats, forming interconnected, dynamic communities. Elucidating the principles of microbial community function is a grand challenge for microbiology, and it will entail experiments that engage microbiomes across multiple levels of complexity. For example, community-level hypotheses often require testing at the mechanistic and/or genetic levels, while mechanistic relationships require community-level evaluation to understand their importance in context. In this Perspective, we articulate the need for model microbiome systems that enable experimentation in both community and reductionist frameworks, with an emphasis on understanding the role of specialized metabolites in microbial communities. We consider essential criteria for developing such model microbiome systems and discuss potential future models that address the ecology of specialized metabolism.

## Abstract (precis)

Microbes occupy diverse habitats, forming interconnected, dynamic communities. Elucidating the principles of microbial community function is a grand challenge for microbiology, and it will entail experiments that engage microbiomes across multiple levels of complexity.

## MICROBIOMES AND SPECIALIZED METABOLISM

Microbes have spent roughly the past 4 billion years evolving in the context of interactive communities, surrounded by thousands of other microorganisms. In the past decade, affordable next-generation sequencing has enabled deep profiling of the microbial biodiversity found in a wide array of ecosystems (1). While these studies have given us an appreciation for the complexity of microbial communities, our understanding of how these microbiomes function is only just beginning.

Connecting the fields of community ecology and chemical ecology may prove critical for understanding microbiome function. Within microbiomes, it is thought that microbial specialized metabolites (e.g., polyketides, nonribosomal peptides, and ribosomally synthesized and posttranslationally modified peptides [RiPPs]), also known as natural products, may play a variety of roles ranging from mediators of cooperation to all-out chemical conflict (2). As such, these molecules may shape the composition and/or spatial distribution of organisms in microbial communities (3). Recently, a number of studies have demonstrated that microbial interspecies interactions can trigger the expression of gene clusters for specialized metabolism that remain silent during growth in pure culture (4, 5). While these observations seem to imply that microbes do use these molecules to mediate interactions, they underscore a key deficiency in our overall knowledge of specialized metabolism: we do not understand why microorganisms make these remarkable molecules nor how they employ this chemical repertoire in their natural habitat.

To evaluate the ecological role(s) of these small molecules, we need model systems of intermediate complexity. This is because axenic culturing techniques fall short of mimicking key aspects of microbial habitats, while environmental samples are often too complex to be understood with molecular-level resolution. Ideal model systems will allow researchers to manipulate the microbial community and test hypotheses with statistical power in a controlled laboratory setting, while also capturing a measure of the complexity of the natural environment.

Several natural and synthetic microbiome systems have been developed to address questions surrounding community ecology, like succession, cooperation, and competition (6). For example, cheese rind (7) and oral biofilms (8) are outstanding models for understanding community succession. Other systems have been studied in light of the specialized metabolic potential of their microbial symbionts (e.g., insects [9] and marine invertebrates [10]), although manipulating microbes in these systems at the genetic level remains a key challenge. Other host-microbiome systems (e.g., bobtail squid-*Vibrio fischeri* association [11], mammalian gut [12], and plant rhizosphere [13]) are proving that systems once studied from a host-microbe perspective are valuable from a microbiome perspective as well. With the success of those models in mind, here we highlight a specific need for model systems that connect microbial chemical ecology and community ecology in tractable systems that reflect natural environments. In this Perspective, we consider criteria for model microbiome systems generally and for examining the ecology of specialized metabolism specifically. By combining tools for studying specialized metabolism and community dynamics, such models will bridge chemical ecology and theoretical community ecology, ultimately allowing us to assess the role of natural products in microbiome function.

## CRITERIA FOR IDEAL MODEL ECOLOGICAL SYSTEMS

We view several elements as essential for maximizing the benefit of building model microbiome systems. However, the overarching consideration is that the model system should enable the testing of predictions originating from community ecology and evolutionary biology, while also enabling mechanistic questions to be addressed through genetic manipulation. Of course, all models are approximations, and no model is perfect, but we suggest that the five criteria outlined below may form the beginning of a guide for identifying natural systems of intermediate complexity that may form the basis for ideal microbiome models. In most cases, we imagine that these microbiome systems will be associated with host organisms, and thus we see the host as an integral part of the model system.

### (i) The model should be based on a natural system.

In our view, beginning with a natural microbial community maximizes the likelihood that the interactions we study will be relevant from a real-world perspective. Beyond this, recurring contact between organisms of different species is generally thought to be a prerequisite for coevolution and for cooperation (14). Thus, building a system with microbes that have routinely encountered each other over evolutionary time may diversify the types of interactions observed within the system.

### (ii) The natural system should contain a relatively simple microbial community.

Most habitats harbor microbial communities with very high species diversity and functional complexity. The mammalian gut and soil are key examples, and while these are among some of the most important systems from a human perspective, their complexity makes them refractory to reductionist experimental regimes. However, other host-associated microbiomes contain communities with a range of complexities. Hosts themselves apply selective pressures that may ultimately define simplified communities relative to microbiomes in environments such as soil. Examples of such host-microbiome communities include the Hawaiian bobtail squid (11), gut communities of nematodes (15) and insects (fruit flies [16] and honeybees [17]), and endophytic root communities (18). Choosing the members that will compose the model community is challenging to do without introducing experimenter bias. For this reason, we favor approaches based on host selection, wherein a natural community can be passaged through its host across several iterations (18). Doing so allows the host to select a reduced community and avoids bias introduced by rational criteria such as phylogeny or functional traits. It is expected that this methodology will afford the simplest functional community to a given host.

### (iii) The model system should have an easily detectable output that indicates healthy microbiome function.

In cases where the model microbiome is associated with a host organism, an overall indicator of microbiome function might be easily measurable host phenotypes, such as enhanced growth, pathogen resistance, or other indicators of stress. In cases where there is no associated host, the output can be based on functional outputs of the microbial community (like metabolic processes) that can be measured directly, as well as community dynamics and metabolomic, transcriptomic, and proteomic pattern analyses. All of these outputs are relevant when comparing the model system with the natural environment, potentially allowing a measure of its ecological relevance.

### (iv) The model system should contain microbial members that are culturable and genetically tractable.

**FIG 1:**
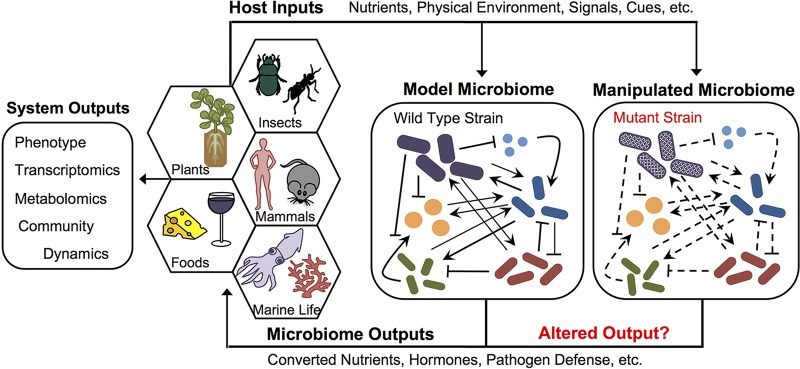
An ideal model system for studying microbiome function may be composed of a community of representative strains originally isolated from a natural source with limited microbial diversity. Genetic manipulation of this microbiome— for example, by substituting for one of the species with an isogenic strain with a targeted gene deletion—will allow for testing of the roles of specific genes/compounds in the microbiome’s function, as well as their influence on microbe-microbe and host-microbiome interactions. This can be evaluated by host phenotypes and global transcriptomics and/or metabolomics or by assessing community dynamics.

Ultimately, a model system must allow for strains with targeted mutations to be substituted for wild-type strains within the community, allowing hypothesis testing at the granular level of genes (Fig. 1). Thus, a model system must contain organisms that are both culturable and genetically tractable, with a particular emphasis on keystone species. While genetic manipulation can be challenging, new techniques for genome editing via CRISPR-Cas9 (clustered regularly interspaced short palindromic repeats with Cas9) (19) and Argonaute (20) offer new opportunities in this regard. Finally, having genome sequences of all community members is key in terms of providing insights regarding the competence of the members and in facilitating targeted genetic analyses.

### (v) The model system should be readily recapitulated in a laboratory setting and scalable.

An ideal model system must be amenable to repeated iteration in a laboratory setting. To fully maximize this aspect of the model, the community should reproducibly reassemble, and the host-community system should be physically small and easily recapitulated on an order of tens to thousands to allow high-throughput interrogation. Moreover, easy reproducibility is essential to allow for quantitative/statistical analyses.

It is unlikely that any single natural system can meet all of the criteria above, but many candidate systems might meet a subset of these criteria. This leads to an important question as researchers seek to evaluate different potential model systems: How can we tell if the model system captures important features of the natural system? This question is closely tied to the third criterion, wherein we suggest that an easily detectable indicator of microbiome health is ideal. In our view, a strong model microbiome will contain the minimum number of strains needed to recapitulate broad patterns seen in the natural system, while meeting as many of the criteria described above as possible.

The above criteria are meant as general considerations for any model microbiome system. However, additional criteria might be considered for models to address specific ecological questions. For example, given our interest in the ecology of specialized metabolites we add the following.

### (i) Microbially produced specialized metabolites should be detectable *in situ*.

Since we seek to understand how specialized metabolites function in microbial communities, we view the ability to detect these molecules *in situ* as a critical aspect of a model system for chemical ecology. Thus, a key step is prior metabolomic investigation to assess the chemical diversity associated with a potential system. Emerging analysis techniques such as molecular networking and chemical dereplication (21) can help researchers rapidly assess such chemical diversity and identify potentially important specialized metabolites with more speed and accuracy than ever before. While measurement of specialized metabolites in natural habitats has been difficult historically, new tools such as mass spectrometry imaging (MSI) and nanospray desorption ionization (nano-DESI) mass spectrometry are providing new opportunities in this regard (22). Beyond this, combining MSI and microscopy will be essential to understand the spatial structure of the community and how it is affected by specialized metabolites.

### (ii) The model community should possess robust specialized metabolic potential.

Once the chemical diversity of a potential system has been evaluated, an important goal is to capture as much of the chemical diversity as possible through inclusion of key community members that contribute to this diversity. This can be achieved based on culturing approaches, where isolates are grown under different conditions—ideally as similar as possible to the natural setting. The chemical profiles of these cultures can then be mapped against the molecular network obtained by the *in situ* analysis. This approach allows for inclusion of isolates that contribute the most to the specialized metabolite diversity detected *in situ*. Alternatively, the specialized metabolic potential could be assessed through genome sequencing of the isolates followed by genome-level analysis using tools like PRISM (23) and antiSMASH (24). Understanding the metabolic profile of the natural environment and how it changes over time will give valuable insights into the chemical ecology of the microbial specialized metabolism. The correlation between community dynamics and metabolomic patterns over time will be key to understanding how these molecules function in microbial communities.

## POTENTIAL MODEL SYSTEMS AND THEIR ANALYSIS

Filamentous actinobacteria (known as actinomycetes) have historically been an important source of useful specialized metabolites. Their extensive specialized metabolisms and genetic tractability make these organisms particularly interesting from the standpoint of chemical ecology. These organisms have traditionally been isolated from soil, which hosts an extremely diverse microbial community. More recently, it has become clear that actinomycetes are broadly associated with plants and insects (25), which widens the possible range of model systems beyond the challenging environment of the soil. The associations in which attine ants and wasps utilize actinomycetes as a means to potentially repel pathogens are notable from the standpoint of chemical ecology, since the hypothetical role of these molecules in these systems is clear. Systems like these may represent a starting point for functional analysis of specialized metabolism *in situ*; however, manipulating the associated microbial community members and recapitulating these systems in a scalable format remain a challenge.

In our own studies, we are exploring microbial communities associated with root nodules of legume plants and with semisocial beetles. This has entailed broadly characterizing these communities using 16S amplicon sequencing, metabolomics analysis for specialized metabolites produced *in situ*, and culturing a wide variety of isolates. Key next steps will be assembling model host-microbiome communities, verifying patterns of succession within these model systems, and identifying specialized metabolites within these systems. Ultimately, we aim to understand the advantages conferred by producing specialized metabolites for the organisms that make them and the role these molecules play in mediating competition and/or cooperation between microbes *in situ*. Importantly, these models will enable investigation of these chemical interactions at the genetic level.

As microbiome science progresses, the development of model microbiome systems that retain the defining features of natural communities will be essential for elucidating the factors that dictate microbiome function. The criteria we outline here are aimed at developing model systems of intermediate complexity that will enable hypothesis testing at the genetic level and interpretation of results in the context of ecological and evolutionary theory. In turn, these results will enable the construction of predictive frameworks that will set the stage for rational manipulation of microbial communities associated with human health, agriculture, and industry.
